# Pain From a Distance: Can Third-Person Self-Talk Mitigate Pain Sensitivity and Pain Related Distress During Experimentally Induced Pain?

**DOI:** 10.1177/00332941241269520

**Published:** 2024-08-01

**Authors:** Helena Gunnarsson, Jens Agerström

**Affiliations:** Department of Psychology, Faculty of Health and Life Sciences, 4180 Linnaeus University, Sweden

**Keywords:** Third-person self-talk, pain, self-distancing, experimental pain, abstract thinking

## Abstract

Pain is self-immersive, leading to a narrow, egocentric focus on the self in the here and now. Preliminary evidence suggests that distancing oneself from the pain can reduce experimentally induced pain. The primary aim of this experimental study was to examine whether a hitherto unexplored, simple self-distancing strategy – “third-person self-talk” – has an analgesic effect on physiological and psychological pain variables. Participants (*N* = 292) were randomly assigned to one of four conditions (third-person self-talk, first-person self-talk, and two control conditions). Pain was induced with a cold pressor apparatus and pain tolerance, pain intensity, negative affect and blood pressure were measured for each group. While in pain, participants engaged in strategic self-talk aided by cue-cards. Data were analyzed with univariate planned comparisons. Few significant differences emerged for the third-person self-talk versus the other conditions. It is concluded that third-person self-talk does not seem to have a meaningful effect on physiological and psychological pain variables, although a small effect size could not be ruled out. Practical implications are discussed.

The study was registered at ClinicalTrials.gov with the ClinicalTrials.gov ID NCT05511857.

Daily living is affected in many chronic pain sufferers (Breivik et al., 2006). When in pain, humans tend to adopt a narrow, egocentric focus on the self (Agerström et al., 2019; Loewenstein, 1996). Such a self-immersed perspective in the here and now is adaptive for survival since pain is a warning signal of tissue damage needing attention (Crombez et al., 2008; Loewenstein, 1996). On the other hand, increasing attention to the pain may heighten its perceived intensity (McCracken, 1997) and negative affect which coexist with painful experiences (Brown et al., 2002; Suhr & Spickard, 2012; Vlaeyen & Linton, 2000). Negative affect tends to be more pronounced in clinical pain but contributes to the suffering part in experimentally induced pain, as well (Bustan et al., 2018).

As human beings we can transcend the self-immersed perspective, which tends to be the default perspective in human perception (Nigro & Neisser, 1983), by adopting a more distant view of ourselves (Trope & Liberman, 2010). So called “third-person self-talk” is one established means of achieving a distanced perspective (Kross et al., 2014; Latinjak et al., 2023; Orvell et al., 2021). Third-person self-talk involves overt or covert verbalizations directed at the self, using one’s own name and second or third-person singular pronouns (e.g., “Jens is feeling happy?”; Latinjak et al., 2023; Orvell et al., 2021). When people use third-person self-talk, they adopt a perspective akin to that of a distanced observer, whereby thinking becomes similar to how one thinks about others. Because it is easier to reason calmly about other people’s emotions than one’s own, emotional reactivity is lower during third-person self-talk (Moser et al., 2017). This might be beneficial when dealing with pain-related distress. Another benefit could be a reduction of people’s attention to the pain itself, which should decrease its perceived intensity (McCracken, 1997), making it more disembodied (Wang et al., 2019). Additionally, third-person self-talk could lower pain-related distress and thereby decrease the discomforting nature in the pain experience (Wall, 1999). Third-person self-talk has been found to diminish the impact of emotional experiences in several contexts (public speech, dating, anxiety-eliciting events) (Kross et al., 2014, 2017; Nook et al., 2017; Streamer et al., 2017), and to reduce emotional reactivity when people reflect on numerous negative experiences (Orvell, 2021). Whether third-person self-talk (e.g., “Helena is feeling pain”), as opposed to first-person self-talk (e.g., I am feeling pain”), impacts physiological or psychological pain variables is unknown.

Although not examining the impact of third-person self-talk, a recent laboratory study provides some preliminary support for the broader idea that self-distancing can have an analgesic effect on some experimental pain dimensions (Wang et al., 2019). Participants who maintained a self-distanced perspective by adopting a “fly on the wall” perspective while experiencing experimentally induced pain reported significantly lower perceived pain intensity and perceived pain stress, compared to self-immersed participants and participants in the control group, but no effect on pain tolerance was found. The results should be interpreted with caution due to the small sample size (∼20 participants per condition in a between-subjects experiment).

The aim of this study was to examine the effect of third-person self-talk on physiological and psychological pain variables. As with much self-talk intervention research, we examine the impact of ‘strategic’ self-talk, whereby people are instructed to use cue words or self-talk scripts developed strategically to facilitate dealing with a difficult situation or achieve performance-related outcomes, and not ‘organic’ self-talk which occurs naturally and spontaneously (see e.g., Latinjak et al., 2023). It was hypothesized that engaging in third-person self-talk would increase pain tolerance, decrease reported pain intensity, negative affect, and blood pressure compared to first-person self-talk, and control group participants.

## Methods

### Power Analysis

Using G*Power an á priori power analysis was performed to determine the sample size. With 85% power, a moderate effect size (f = .25), and a conventional significance level of *p* < .05 (two-tailed), a total of 292 participants (73 participants in each group) were required for univariate planned comparisons.

### Participants

In the experiment, 292 students and staff (107 males, 185 females) at a university in Sweden participated. Inclusion criteria were 18 years of age or older, sufficient knowledge in Swedish language. Exclusion criteria were known disease affecting cognitive abilities (e.g., dementia, multiple sclerosis), and known disease affecting the peripheral or central nervous system (e.g., present clinical pain, multiple sclerosis, polyneuropathy). The final sample consisted of 292 eligible participants, randomly assigned to four different groups (third-person self-talk (*n* = 73, 28 males, 45 females, age range = 18–43 years; mean age 25 years); first-person self-talk (*n* = 73, 25 males, 48 females, age range = 18–41 years, mean age 25 years); cue-card control condition (*n* = 73, 29 males, 44 females, age range = 19–48 years, mean age 25 years); non-interventional control condition (*n* = 73, 25 males, 48 females, age range = 19–66 years, mean age 25 years). The study was approved by the Swedish Ethical Review Authority (number: 2022-02,423-01) and was conducted in accordance with the ethical standards of the 1964 Helsinki Declaration. All participants signed an informed consent form.

### Experimental Pain Induction

In this experiment, pain was induced by a cold pressor apparatus (Nuve BM-302 Milmedtek AB, Nättraby, Sweden). The cold pressor apparatus contained 10 L of circulating water at a temperature of 1^0^ C in this experiment. The cold pressor apparatus is a well-known method of pain induction (Agerström et al., 2019; Dawson & List, 2009; Dowman et al., 2008; Hines & Brown, 1936; Neziri et al., 2011; Sharpe et al., 2015; Stening et al., 2007). During the intervention the participant was asked to keep their left hand immersed in the cold water up to processus styloideus radii/ulnae for as long as possible. The maximum time to keep the hand immersed in the cold water was 5 minutes. If the participant had not withdrawn their hand after 5 minutes, the experimenter instructed the participant to remove the hand from the cold water.

### Dependent Variables

#### Pain Intensity

A numerical rating scale (NRS) was used to measure the experimentally induced pain during the test session. The NRS reaches from zero (no pain) to 10 (most intense pain imaginable). The participants would report the estimated level of their pain orally immediately after withdrawing their hand from the cold water-bath while looking at a NRS held by the experimenter. The NRS is a unidimensional, pain-intensity scale which can be influenced by other factors than the pain intensity itself. Ratings show high correlations with the Visual Analogue Scale (VAS) that is also commonly used to measure pain intensity. The NRS has been shown to correlate very strongly (r = .93) with measurements from the VAS, but in comparison the NRS have been reported to be more sensitive (Ferreira-Valente et al., 2011). Both the NRS and the VAS are considered valid measures of pain intensity (Ferreira-Valente et al., 2011; Thong et al., 2018).

#### Pain-Related Negative Affect

The NRS-A (Numerical Rating Scale – Anxiety) was used to measure the pain-related negative affect (worry, fear) experienced during the pain induction. The NRS-A reaches from zero (not at all) to 10 (worst imaginable). The participants would report the estimated level of their (worry, fear) immediately after the pain estimation on the NRS. An earlier study has concluded a high correlation between the NRS-A and STAI (State-Trait Anxiety Inventory) (r = .778–.807) (Propokowicz et al., 2022).

#### Pain Tolerance

Pain tolerance was measured as the time in seconds each participant withheld their hand in the cold water-bath.

#### Blood Pressure

Blood pressure was measured using an Omron automatic upper arm blood pressure monitor (HEM-9210T). Blood pressure measurements were conducted twice during the test session, one baseline measurement and one measurement during each intervention, while the participant was simultaneously induced with pain. The difference between the two measurements were used in the statistical analysis. The cold pressor test induces a vascular sympathetic response when the hand is immersed in cold water (Mourot et al., 2009).

#### Interventions

The four different conditions were third-person self-talk (self-distanced view); first-person self-talk (self-immersed view); cue-card control; non-intervention control. Participants in the third-person self-talk condition received a cue-card containing nine pain-related sentences with the participant’s first name. An example of a sentence was “Helena’s pain is transmitted through signaling in the nervous system”. Participants in the first-person self-talk condition received a cue-card containing the same nine pain-related sentences but with “I” and “My” as pronouns. e.g., “My pain is transmitted through signaling in the nervous system”. Thus, the manipulation concerned strategic self-talk rather than organic self-talk. The cue-card control condition consisted of a cue-card with the same sentences about pain but that were neither self-distanced nor self-immersed. e.g., “The pain is transmitted through signaling in the nervous system”. This was to control for any effect produced by the cue-card itself (e.g., distraction). In the non-interventional control condition, the participants did not receive any cue card, nor were they induced with a specific perspective.

#### Manipulation Check

To ascertain that the participants were subjected to the experimental manipulation, they were asked to estimate how well they had adopted the perspective described on the cue-card on a Likert – scale. This estimation was conducted twice, after their practice session and after their intervention session. The Likert – scale ranged from 0-7, where zero equaled “not at all” and 7 equaled “all the time”.

### Procedure

Participants were introduced to a study on how different thought processes could influence pain perception. They then read the information pamphlet intended for research participants thoroughly and the experimenter answered any questions about the experiment. All participants provided informed consent before participating in the experiment. The experimenter provided standardized written and oral instructions to all participants during the entire test session. At the start of the test session the participant was comfortably seated in a chair next to the cold pressor apparatus. A blood pressure cuff was placed around the upper right arm, and when the participant had been in a sitting posture for 5 minutes a baseline blood pressure measurement was conducted. Next, the participants were presented with one of the four interventions on a computer screen in front of them. They were instructed to silently read and think according to the sentences on one of the presented cue-cards (third-person self-talk; first-person self-talk; cue-card control) for 2 minutes, and a timer was set to measure the time. Participants assigned to the (non-interventional control condition) were not instructed to adopt a specific perspective. Instead, they received the information that they could think about whatever they preferred for 2 minutes. After 2 minutes the participants reading one of the cue-cards were asked to estimate their compliance in following the sentences on the cue-card on a Likert – scale (manipulation check). After that, the participants received written instructions to try to withhold their left hand in the cold water-bath for as long as possible or until instructed otherwise, while at the same time reading the cue-card on the screen in front of them. The participants in the control condition had no screen to look at. Before immersing the hand in the cold water-bath, all participants confirmed to the experimenter that they understood the task. The participants immersed the left hand in the cold water-bath up to processus styloideus radii/ulnae at the same time as a timer was started. After 5 seconds with the hand immersed in cold water a second blood pressure measurement was started and the blood pressure measurement was written down. When the participants withdrew their hand from the cold water-bath, they were immediately asked to estimate the experienced pain on the NRS, and then the negative affect on the NRS. The time with the hand immersed in the cold water and the NRS estimates were noted in the protocol. A short debriefing concluded the session. Each participant received a movie ticket for their participation.

### Data analysis

Outliers were detected through z-score analysis, and values above 3.29 were removed. The following dependent measures were not normally distributed, and no transformation resolved the problem: time with the hand immersed in the cold water-bath in seconds, estimated pain (NRS), estimated negative affect (NRS). Non-parametric Mann-Whitney U – tests were used to analyze these measures. The remaining dependent measures were normally distributed and parametric (Student’s) t-tests were used. Planned comparisons were conducted between the third-person self-talk condition versus first-person self-talk condition and versus the two control conditions for each variable. We used uncorrected α – levels because our comparisons were planned, and our á priori power analysis was based on an α-value of .05.

## Results

### Manipulation Check

A paired *t* test was conducted between the practice session and the intervention session regarding the estimated values on the Likert-scale, and a significant difference between the practice session (M = 5.753, SD = 1.127) and the intervention session (M = 5.146, SD = 1.702; t(218) = 4.679, *p* < .001, Cohen’s d = 1.921 was found. The analysis showed that it was easier for the participants to engage in the different self-talk conditions without pain induction compared to when the participants experienced pain. This was expected, since pain tends to limit cognitive resources (Moriarty et al., 2011). However, the mean value for engaging in the task was still high (mean 5.146 with 7 as the maximum value), which means that participants substantially engaged their thoughts in the self-talk task during the pain induction.

### Descriptive Statistics

Mean and SD for the different conditions (Third-person self-talk; First-person self-talk; Cue-card control; Non-interventional Control are presented in Table 1.

**Table 1.** Mean and SD for the Different Conditions (Third-Person Self-Talk; First-Person Self-Talk; Cue-Card Control; Non-interventional Control). For all Blood Pressure Measurements, the Difference Between the Intervention Measure and Baseline Measure is Reported.

Condition	Pain tolerance mean (SD)	Pain intensity (mean, SD)	Negative affect (mean, SD)	Systolic blood pressure (mean, SD)	Diastolic blood pressure (mean, SD)	Pulse (mean, SD)
Third-person self-talk	132.34 (115.1)	7.01 (1.22)	4.52 (2.20)	−11.20 (9.28)	−10.75 (8.79)	−10.86 (11.31)
First-person self-talk	149.47 (118.74)	7.24 (1.26)	4.75 (2.37)	−13.84 (8.83)	−12.21 (8.70)	−11.77 (10.26)
Cue-card control	161.52 (119.71)	7.00 (1.47)	4.97 (2.44)	−10.28 (10.66)	−10.28 (10.66)	−13.16 (12.02)
Control	168.56 (121.27)	6.91 (1.47)	4.33 (2.66)	−12.62 (9.91)	−13.83 (7.91)	−12.64 (13.26)

### Inferential Statistics

#### Pain Tolerance

The third-person self-talk condition did not differ significantly compared to the first-person self-talk condition (U = 2505.00, z = −.634, *p* < .526, r = −.074), the cue-card control condition (U = 2314.50, z = −1.40, *p* < .162, r = −.164), or the non-interventional control condition (U = 2208.00, z = −1.829, *p* < .067, r = −.214). Hence, the hypothesis that third-person self-talk would increase pain tolerance was not supported.

#### Pain intensity

The third-person self-talk condition did not differ significantly compared to the first-person self-talk condition (U = 2335.00, z = −1.317, *p* < .188, r = −.154), the cue-card control condition (U = 2497.50, z = −.524, *p* < .600, r = −.061), or the non-interventional control condition (U = 2577.00, z =−.206, *p* < .837, r = −.024). Thus, the hypothesis that third-person self-talk would lower the perceived pain intensity was not supported.

#### Negative Affect

The third-person self-talk condition did not differ significantly compared to the first-person self-talk condition (U = 2492.00, z = −.680, *p* < .496, r = −.080), the cue-card control condition (U = 2388.50, z = −1.087, *p* < .277, r = −.127), or the non-interventional control condition (U = 2562.00, z = −.404, *p* < .686, r = −.047). Hence, the hypothesis that third-person self-talk would decrease the pain-related negative affect was not supported.

#### Systolic Blood Pressure

The third-person self-talk condition did not differ significantly compared to the first-person self-talk condition (*t*(120) = 1.607, *p* < .111, Cohen’s d = .291), the cue-card control condition (*t*(129) = −.525, *p* < .600, Cohen’s d = −.092), or the non-interventional control condition (*t*(128) = .841, *p* < .402, Cohen’s d = .148). Hence, the hypothesis that third-person self-talk would lower the systolic blood pressure was not supported.

#### Diastolic Blood Pressure

The third-person self-talk condition did not differ significantly compared to the first-person self-talk condition (*t*(120) = .917, *p* < .361, Cohen’s d = .166) or the cue-card control condition (*t*(128) = −.149, *p* < .882, Cohen’s d = −.026). However, the third-person self-talk condition showed significantly lower diastolic blood pressure compared to the non-interventional control condition (*t*(129) = 2.1, *p* < .037), Cohen’s d .368. Overall, the hypothesis that third-person self-talk would lower the diastolic blood pressure received only weak support with the third-person self-talk condition only showing lower blood pressure than the non-interventional control group, but not the first-person or cue-card control conditions.^1^

#### Pulse

The third-person self-talk condition did not differ significantly compared to the first-person self-talk condition (*t*(118) = .462, *p* < .645, Cohen’s d = .084), the cue-card control condition (*t*(125) = −1.110, *p* < .269, Cohen’s d = .197), or the non-interventional control condition (*t*(127) = .818, *p* < .415, Cohen’s d = .144). Hence, the hypothesis that third-person self-talk would lower the pulse was not supported.

#### Pain tolerance and Negative Affect

A correlation analysis confirmed a negative correlation between pain tolerance and negative affect (r = −.283, *p* < .001). This correlation was expected, since a high level of negative affect would mean high perceived discomfort during the pain induction, and hence lower the amount of time the pain could be tolerated.

As a sensitivity analysis, we also performed the analyses excluding the participants estimating below 3 on the manipulation check Likert-scale, but the results remained virtually the same.

## Discussion

The aim of this study was to examine the effect of third-person self-talk (self-distancing perspective) on pain tolerance, pain intensity, pain-related negative affect, and blood pressure in experimentally induced pain in healthy participants. It was hypothesized that engaging in third-person self-talk would lower pain intensity and negative affect, increase pain tolerance, and lower blood pressure. In this study, we did not find any effect of third-person self-talk on pain tolerance, estimated pain intensity, or estimated negative affect. Nor did we find any effect on blood pressure (systolic or diastolic) or the pulse when the third-person self-talk condition was compared to first-person self-talk or the cue-card control condition. When comparing the third-person self-talk to the non-interventional control condition, significantly decreased diastolic blood pressure was found. Considering that no other dependent variables differed significantly between the third-person self-talk condition and any of the other conditions, this difference seems to be of little importance when considering third-person self-talk as a potential pain-relieving strategy. Since this was a well-powered study, and the descriptive data showed that the participants experienced substantial pain induction levels with the cold pressor test (Table 1), and complied with the experimental manipulation, it appears that engaging in self-distancing through third-person self-talk does not have a meaningful effect on the measured experimental pain variables.

Our hypothesis regarding third-person self-talk was partly informed by previous research on the facilitating effects of third-person self-talk on emotion-regulation (Kross et al., 2014, 2017; Nook et al., 2017; Orvell et al., 2021; Streamer et al., 2017) and the coexistence of negative affective states and physical pain (Brown et al., 2002; Suhr & Spickard, 2012; Vlaeyen & Linton, 2000). However, it should be noted that the link between the nociceptive transmission and the negative emotional affective state is much stronger in clinical pain compared to induced experimental pain.

Pain is part of our survival system, like hunger, thirst, sleep, and sex. To avoid pain is highly rewarding (Porreca & Navratilova, 2017), but possibly, the knowledge that we could easily stop the pain, would lower the threatening value coexisting with pain substantially. There is support for the notion that the prefrontal cortex governs the activation in brain areas responsible for the emotional component in pain experiences (Thompson & Neugebauer, 2017; Wiech & Tracey, 2009), and this could possibly affect the threatening value in experimentally induced pain. The induced experimental pain in this study was short in duration and could be terminated whenever the participant wanted. When enrolling into the research experiment the participants were also informed that the pain induction would be completely harmless, and that they could terminate the pain induction whenever they wanted. Therefore, it is likely that the experienced pain was accompanied by lower levels of emotional distress compared to the pain experience in clinical pain. Some support for this could be seen in the descriptive data, where the mean of the negative affective estimations was below the pain intensity measures for all groups (Table 1). The mean estimation of negative affect was close to the midpoint of the scale for all different conditions, and it has been suggested that an estimation of 4–6 on the NRS-A could be categorized as medium-fairly anxious (Propokowicz et al., 2022). It is possible that this level of negative affect was too low to allow for an observable effect of third-person self-talk.

The results from our study were not in line with the results from the laboratory study conducted by Wang et al. (2019), where a self-distanced perspective significantly decreased both pain intensity ratings and ratings of pain stress. However, there are two important differences between Wang’s et al. (2019) study and our study. One is that they did not induce the self-distanced perspective through third-person self-talk. Instead, the participants adopted a self-distanced perspective from a “fly on the wall” vantage point. Perhaps these different induction methods could affect the amount of self-distancing achieved. Third-person self-talk has been used successfully in earlier research to induce a self-distanced perspective, and to lower negative emotional reactivity and reduce the impact of emotional experiences (Kross et al., 2014, 2017; Nook et al., 2017; Orvell et al., 2021; Streamer et al., 2017). Another important difference was that the Wang et al. study (2019) was considerably smaller with 21–23 participants in each experimental (between-subjects) condition (*N* = 65). In their study, the observed self-distancing effect on pain intensity and stress was large. It might, of course, be that Wang et al. estimated the true effect. However, it should be interpreted with caution given that small sample sizes tend to yield more variable effect sizes, reducing the likelihood that a statistically significant result reflects a true effect (Button et al., 2013).

The results from this study are consistent with the results from an earlier, well-powered experimental study investigating the effect of abstract thinking on experimental pain (Gunnarsson & Agerström, 2023). In that study, the participants adopted an abstract mindset (*why* they experienced the experimental pain) or a concrete mindset (*how* they experienced the experimental pain). Because a third-person perspective is more psychologically distant and abstract than a first-person perspective (Kross et al., 2014; Trope & Liberman, 2010) the results converge at a conceptual level.

The results of the current study suggest that engaging in third-person self-talk has no effect on acute, experimental pain. The practical implication of this finding is that is seems futile for people to use third-person self-talk to alleviate acute pain, although there is nothing in our data suggesting that it has a debilitating effect. It is, of course, possible that we failed to detect a small effect since our study only had high a priori power to detect a medium-sized effect. However, whether a small effect would be meaningful in the current context could be debated. Furthermore, as a general observation, it seems rare for experimental studies on pain management to be adequately powered to detect small effects.

Future research should examine the effectiveness of educational self-talk interventions performed outside the laboratory, such as in the home or at work, by people with chronic pain. Importantly, such interventions should be designed to elicit organic self-talk, and hence to increase chronic pain patients’ inclination to spontaneously use third-person self-talk in their everyday lives. Besides making the occurrence of self-talk more natural, this type of self-talk may facilitate the participants’ rational thought processes, possibly having a pain-relieving effect. Generally speaking, third-person self-talk should tax relatively few cognitive control resources (Moser et al., 2017), which is arguably important since chronic pain is very resource depleting (Berryman et al., 2013; Legrain et al., 2009; Nouwen et al., 2006). Compared to other available psychological treatment programs of chronic pain, such as cognitive behavioral therapy (CBT) and acceptance and commitment therapy (ACT), which tend to be lengthy and require extensive training with a clinician (McCracken et al., 2022; Williams et al., 2020), third-person self-talk may be easier to learn and conduct at home whenever needed.

It is also possible that other types of self-distancing techniques, such as the “fly on the wall” perspective adopted by participants in the Wang et al. (2019) study, are more effective although larger studies are needed to establish this. Future research also needs to examine whether such self-distancing techniques have the potential to alleviate *chronic* pain and pain related distress. We believe that self-distancing could be a viable strategy when dealing with chronic pain given the more prominent role that negative emotions (e.g., fear and anxiety) play in chronic pain (Brown et al., 2002; Suhr & Spickard, 2012; Vlaeyen & Linton, 2000) and the robust finding that self-distancing reduces emotional reactivity and the impact of emotional experiences across numerous contexts (Kross et al., 2014, 2017; Nook et al., 2017; Orvell et al., 2021; Streamer et al., 2017).

Our experiment comes with some limitations. The fact that participants knew their pain would be brief and that they could terminate it at their own discretion meant that negative affect was kept relatively low. Hence, there was relatively little emotional reactivity to regulate in the first place, which may have restricted the impact of third-person self-talk. In addition, although the experiment included two control conditions (one controlling for a possible cue card effect and another eliciting no specific strategy), there was no comparison strategy for third-person self-talk other than first-person self-talk. It would have been interesting to include another comparison condition consisting of a successful pain management strategy (e.g., distraction) as a benchmark. However, this would have been more useful had we found an effect of third person self-talk whose effectiveness we could compare to more established strategies. In the non-interventional control condition, where the participants were allowed to think of whatever they preferred, we had no manipulation check to shed light on any potentially pain-relieving strategy they might have used. It is possible that some participants in this condition might have spontaneously engaged in self-distancing practices, such as imagining themselves being outside of their bodies, or imagining themselves being somewhere else, or even using organic self-talk. This could be seen as a limitation in our study. Hence, future experimental studies examining the effectiveness of self-distancing and third person self-talk should control for spontaneous use of such strategies among non-intervention participants. In addition, it would also be interesting to examine how often pain-afflicted individuals spontaneously use such pain management strategies in everyday life using diary methods.

Another limitation of the current study is that the experimenter was not blinded to the different interventions. However, to counteract experimenter bias and demand characteristics, the experimenter provided standardized written and oral instructions to all participants. Therefore, we believe it is unlikely that any systematic bias would have impacted the results.

Finally, it should be noted that although the theoretical rationale underlying our hypothesis was partly based on the assumption that attention to pain (facilitated by a concrete self-immersed first-person perspective) would increase its perceived intensity (e.g., McCracken, 1997), the impact of attention needs clarification. There are studies suggesting that pain intensity is dynamically modulated by attention (Adamczyk et al., 2023; Kucyi & Davis, 2015; Quevedo & Coghill, 2007). For example, when attention is focused on a more restricted (as opposed to global) body area of induced pain, perceived pain intensity is reduced (Adamczyk et al., 2023). Although this is an interesting finding that paints a somewhat more complex picture of attention, we do not see this as conflicting with our theoretical rationale which simply assumes that a self-immersed first-person perspective would increase attention to the pain per se. Nevertheless, future research may want to examine whether first-person and third-person self-talk influence perceived pain intensity differently depending on whether the pain is localized as opposed to more global (expanding across body areas).

In conclusion, this relatively large and well-powered experimental study showed no effect of third-person self-talk on pain tolerance, pain intensity, pain-related negative affect, or blood pressure (systolic, diastolic, pulse) in conjunction with experimentally induced pain. Since clinical pain is different from experimental pain, future research should examine whether self-distancing, and third-person self-talk specifically, has the potential to alleviate clinical pain states, especially chronic pain states.

## Note

1. With Bonferroni correction, this result became non-significant.

## References

[bibr1-00332941241269520] Adamczyk W. M. Katra M. Szikszay T. M. Peugh J. King C. D. Luedtke K. Coghill R. C. (2023). Spatial tuning in nociceptive processing is driven by attention. The Journal of Pain, 24(6), 1116–1125. 10.1101/2022.06.16.496352 36965648 PMC10330125

[bibr3-00332941241269520] Agerström J. Stening K. Axman O. (2019). Pain here and now: Physical pain impairs transcendence of psychological distance. Journal of Pain Research, 12, 961–968. 10.2147/JPR.S194114 30881106 PMC6417848

[bibr4-00332941241269520] Berryman C. Stanton T. R. Jane Bowering K. Tabor A. McFarlane A. Lorimer Moseley G. (2013). Evidence for working memory deficits in chronic pain: A systematic review and meta-analysis. Pain, 154(8), 1181–1196. 10.1016/j.pain.2013.03.002 23707355

[bibr5-00332941241269520] Breivik H. Collett B. Ventafridda V. Cohen R. Gallacher D. (2006). Survey of chronic pain in europe: Prevalence, impact on daily life, and treatment. European Journal of Pain, 10(4), 287–333. 10.1016/j.ejpain.2005.06.009 16095934

[bibr6-00332941241269520] Brown S. C. Glass J. M. Park D. C. (2002). The relationship of pain and depression to cognitive function in rheumatoid arthritis patients. Pain, 96(3), 279–284. 10.1016/S0304-3959(01)00457-2 11973000

[bibr7-00332941241269520] Bustan S. Gonzalez-Roldan A. M. Schommer C. Kamping S. Loffler M. Brunner M. Flor H. Anton F. (2018). Psychological, cognitive factors and contextual influences in pain and pain-related suffering as revealed by a combined qualitative and quantitative assessment approach. PLoS One, 13(7), Article e0199814. 10.1371/journal.pone.0199814 30063704 PMC6067693

[bibr8-00332941241269520] Button K. Ioannidis J. Mokrysz C. Nosek B. A. Flint J. Robinson E. S. J. Munafò M. R. (2013). Power failure: why small sample size undermines the reliability of neuroscience. Nature Reviews Neuroscience, 14(5), 365–376. 10.1038/nrn3475 23571845

[bibr10-00332941241269520] Crombez G. Eccleston C. De Vlieger P. Van Damme S. De Clercq A. (2008). Is it better to have controlled and lost than never to have controlled at all? An experimental investigation of control over pain. Pain, 137(3), 631–639. 10.1016/j.pain.2007.10.028 18063311

[bibr11-00332941241269520] Dawson A. List T. (2009). Comparison of pain thresholds and pain tolerance levels between Middle Easterners and Swedes and between genders. Journal of Oral Rehabilitation, 36(4), 271–278. 10.1111/j.1365-2842.2009.01943.x 19220713

[bibr12-00332941241269520] Dowman R. Rissacher D. Schuckers S. (2008). EEG indices of tonic pain-related activity in the somatosensory cortices. Clinical neurophysiology: Official Journal of the International Federation of Clinical Neurophysiology, 119(5), 1201–1212. 10.1016/j.clinph.2008.01.019 18337168 PMC2676940

[bibr13-00332941241269520] Ferreira-Valente M. A. Pais-Ribeiro J. L. Jensen M. P. (2011). Validity of four pain intensity rating scales. Pain, 152(10), 2399–2404. 10.1016/j.pain.2011.07.005 21856077

[bibr46-00332941241269520] Gunnarsson H. Agerström J. (2023). Thinking abstractly about one’s physical pain: can abstraction reduce sensitivity to painful stimuli? Nordic Psychology, 76(1), 134–145.

[bibr14-00332941241269520] Hines E. A. Brown G. E. (1936). The cold pressor test for measuring the reactibility of the blood pressure: Data concerning 571 normal and hypertensive subjects. American Heart Journal, 1(11), 1–9. 10.1016/s0002-8703(36)90370-8

[bibr15-00332941241269520] Kross E. Bruehlman-Senecal E. Park J. Burson A. Dougherty A. Shablack H. Bremner R. Moser J. Ayduk Ö. (2014). Self-talk as a regulatory mechanism: How you do it matters. Journal of Personality and Social Psychology, 106(2), 304–324. 10.1037/a0035173 24467424

[bibr16-00332941241269520] Kross E. Vickers B. D. Orvell A. Gainsburg I. Moran T. P. Boyer M. Jonides J. Moser J. Ayduk O. (2017). Third person selftalk reduces Ebola worry and risk perception by enhancing rational thinking. Applied Psychololgy: Health and Well-Being, 9(3), 387–409. 10.1111/aphw.12103 29171194

[bibr17-00332941241269520] Kucyi A. Davis K. D. (2015). The dynamic pain connectome. Trends in Neurosciences, 38(2), 86–95. 10.1016/j.tins.2014.11.006 25541287

[bibr18-00332941241269520] Latinjak A. T. Morin A. Brinthaupt T. M. Hardy J. Hatzigeorgiadis A. Kendall P. C. Neck C. Oliver E. J. Puchalska-Wasyl M. M. Tovares A. V. Winsler A. (2023). Self-Talk: An interdisciplinary review and transdisciplinary model. Review of General Psychology, 27(4), 355–386. 10.1177/10892680231170263

[bibr19-00332941241269520] Legrain V. Damme S. V. Eccleston C. Davis K. Seminowicz D. Crombez G. (2009). A neurocognitive model of attention to pain: Behavioral and neuroimaging evidence. Pain, 144(3), 230–232. 10.1016/j.pain.2009.03.020 19376654

[bibr20-00332941241269520] Loewenstein G. (1996). Out of control: Visceral influences on behavior. Organizational Behavior and Human Decision Processes, 65(3), 272–292. 10.1006/obhd.1996.0028

[bibr21-00332941241269520] McCracken L. M. (1997). Attention” to pain in persons with chronic pain: A behavioral approach. Behavior Therapy, 28(2), 271–284. 10.1016/S0005-7894(97)80047-0

[bibr22-00332941241269520] McCracken L. M. Yu L. Vowles K. E. (2022). New generation psychological treatments in chronic pain. BMJ, 2022; 376, e057212. 10.1136/bmj-2021-057212 35228207

[bibr23-00332941241269520] Moriarty O. McGuire B. E. Finn D. P. (2011). The effect of pain on cognitive function: A review of clinical and preclinical research. Progress in Neurobiology, 93(3), 385–404. 10.1016/j.pneurobio.2011.01.002 21216272

[bibr24-00332941241269520] Moser J. S. Dougherty A. Mattson W. Katz B. Moran T. P. Guevarra D. Shablack H. Ayduk O. Jonides J. Berman M. G. Kross E. (2017). Third-person self-talk facilitates emotion regulation without engaging cognitive control: Converging evidence from ERP and fMRI. Scientific Reports, 7(1), 4519. 10.1038/s41598-017-04047-3 28674404 PMC5495792

[bibr25-00332941241269520] Mourot L. Bouhaddi M. Regnard J. (2009). Effects of the cold pressor test on cardiac autonomic control in normal subjects. Physiological Research, 58(1), 83–91. 10.33549/physiolres.931360 18198985

[bibr26-00332941241269520] Neziri A. Y. Scaramozzino P. Andersen O. K. Dickenson A. H. Arendt-Nielsen L. Curatolo M. (2011). Reference values of mechanical and thermal pain tests in a pain-free population. European Journal of Pain, 15(4), 376–383. 10.1016/j.ejpain.2010.08.011 20932788

[bibr27-00332941241269520] Nigro G. Neisser U. (1983). Point of view in personal memories. Cognitive Psychology, 15(4), 467–482. 10.1016/0010-0285(83)90016-6

[bibr28-00332941241269520] Nook E. C. Schleider J. L. Somerville L. H. (2017). A linguistic signature of psychological distancing in emotion regulation. Journal of Experimental Psychology: General, 146(3), 337–346. 10.1037/xge0000263 28114772

[bibr29-00332941241269520] Nouwen A. Cloutier C. Kappas A. Warbrick T. Sheffield D. (2006). Effects of focusing and distraction on cold pressor–induced pain in chronic back pain patients and control subjects. The Journal of Pain, 7(1), 62–71. 10.1016/j.jpain.2005.08.004 16414557

[bibr30-00332941241269520] Orvell A. Vickers B. D. Drake B. Verduyn P. Ayduk O. Moser J. Jonides J. Kross E. (2021). Does distanced self-talk facilitate emotion regulation across a range of emotionally intense experiences? Clinical Psychological Science, 9(1), 68–78. 10.1177/2167702620951539

[bibr31-00332941241269520] Porreca F. Navratilova E. (2017). Reward, motivation and emotion of pain and its relief. Pain, 158(Suppl 1), S43–S49. 10.1097/j.pain.0000000000000798 28106670 PMC5350036

[bibr32-00332941241269520] Prokopowicz A. Stanczykiewicz B. Uchmanowicz I. (2022). Validation of the numerical anxiety rating scale in postpartum females: A prospective observational study. Ginekologia Polska, 93(9), 686–694. 10.5603/GP.a2021.0197 35072220

[bibr33-00332941241269520] Quevedo A. S. Coghill R. C. (2007). Attentional modulation of spatial integration of pain: Evidence for dynamic spatial tuning. Journal of Neuroscience: The Official Journal of the Society for Neuroscience, 27(43), 11635–11640. 10.1523/JNEUROSCI.3356-07.2007 17959806 PMC6673211

[bibr34-00332941241269520] Sharpe L. Johnson A. Dear B. F. (2015). Attention bias modification and its impact on experimental pain outcomes: Comparison of training with words versus faces in pain. European Journal of Pain, 19(9), 1248–1257. 10.1002/ejp.648 25523240

[bibr35-00332941241269520] Stening K. Eriksson O. Wahren L. Berg G. Hammar M. Blomqvist A. (2007). Pain sensations to the cold pressor test in normally menstruating women: Comparison with men and relation to menstrual phase and serum sex steroid levels. American Journal of Physiology - Regulatory, Integrative and Comparative Physiology, 293(4), R1711–R1716. 10.1152/ajpregu.00127.2007 17652363

[bibr36-00332941241269520] Streamer L. Seery M. D. Kondrak C. L. Lamarche V. M. Saltsman T. L. (2017). Not I, but she: The beneficial effects of self-distancing on challenge/threat cardiovascular responses. Journal of Experimental Social Psychology, 70(5), 235–241. 10.1016/j.jesp.2016.11.008

[bibr37-00332941241269520] Suhr J. Spickard B. (2012). Pain-related fear is associated with cognitive task avoidance: Exploration of the cogniphobia construct in a recurrent headache sample. Clinical Neuropsychologist, 26(7), 1128–1141. 10.1080/13854046.2012.713121 22928643

[bibr38-00332941241269520] Thompson J. M. Neugebauer V. (2017). Amygdala plasticity and pain. Pain Research and Management, *2017* , 1–12. Article ID 8296501. 10.1155/2017/8296501 PMC5742506 29302197

[bibr39-00332941241269520] Thong I. S. K. Jensen M. P. Miro J. Tan G. (2018). The validity of pain intensity measures: What do the NRS, VAS, VRS and FPS-R measure? Scandinavian journal of pain, 18(1), 99–107. 10.1515/sjpain-2018-0012 29794282

[bibr40-00332941241269520] Trope Y. Liberman N. (2010). Construal-level theory of psychological distance. Psychological Review, 117(2), 440–463. 10.1037/a0018963 20438233 PMC3152826

[bibr41-00332941241269520] Vlaeyen J. W. S. Linton S. J. (2000). Fear-avoidance and its consequences in chronic musculoskeletal pain: A state of the art. Pain, 85(3), 317–332. 10.1016/S0304-3959(99)00242-0 10781906

[bibr42-00332941241269520] Wall P. (1999). Pain: The science of suffering. Gurugram: Orion.

[bibr43-00332941241269520] Wang T. Yang L. L. Yang Z. Huang X. T. (2019). Imagining my painful hand is not mine: Self-distancing relieves experimental acute pain induced by a cold pressor task. The Journal of Pain, 20(3), 358–365. 10.1016/j.jpain.2018.10.001 30339929

[bibr44-00332941241269520] Wiech K. Tracey I. (2009). The influence of negative emotions on pain: Behavioral effects and neural mechanisms. NeuroImage, 47(3), 987–994. 10.1016/j.neuroimage.2009.05.059 19481610

[bibr45-00332941241269520] Williams A. C. Fisher E. Hearn L. Eccleston C. (2020). Psychological therapies for the management of chronic pain (excluding headache) in adults. The Cochrane Database of Systematic Reviews, 8, Article CD007407. 10.1002/14651858.CD007407.pub4 PMC7437545 32794606

